# The feeling, embodiment and emotion of hallucinations in first episode psychosis: A prospective phenomenological visual-ecological study using novel multimodal unusual sensory experience (MUSE) maps

**DOI:** 10.1016/j.eclinm.2021.101153

**Published:** 2021-10-16

**Authors:** Katie Melvin, Jon Crossley, John Cromby

**Affiliations:** a University of Leicester, Department of Neuroscience, Psychology and Behaviour, George Davies Centre, University of Leicester, 15 Lancaster Road, Leicester, LE1 7HA; b Leicestershire Partnership National Health Service (NHS) Trust, George Davies Centre, University of Leicester, 15 Lancaster Road, Leicester, LE1 7HA; c University of Leicester, Division of Innovation, Technology and Operations, Brookfield Campus, 266 London Road, Leicester, LE2 1RQ

**Keywords:** Hallucination, Methods, Early Intervention in Psychosis, Psychosis, Schizophrenia, Body-map, Feeling, Emotion, Embodiment, Visual, Ecological, Qualitative

## Abstract

**Background:**

Research and practice typically focus upon unimodal hallucinations, especially auditory verbal hallucinations. Contemporary research has however indicated that voice-hearing may co-occur within a broader milieu of feelings, and multimodal hallucinations may be more common than previously thought.

**Methods:**

An observational design asked participants to prospectively document the feeling and modality of hallucinations for one week prior to an interview. Novel visual diary methods involving drawing, writing and body-mapping generated 42 MUSE maps (multimodal unusual sensory experience), analysed with a participatory qualitative method. Twelve people took part: all experiencing hallucinations daily, accessing early intervention in psychosis services, given psychotic-spectrum diagnoses, and living in the community. The study took place during a seven-month period in 2018 at Leicestershire and Rutland's Psychosis Intervention and Early Recovery service (UK).

**Findings:**

All documented hallucinations co-occurred with bodily feelings. Feelings were localised to specific body areas, generalised across the body and extended beyond the body into peripersonal space. Co-occurring emotional feelings most commonly related to confusion, fear and frustration.

**Interpretation:**

Hallucinations were characterised by numerous feelings arising at once, often including multimodal, emotional, and embodied features. Within this study, the immediate feeling of hallucination experiences were readily communicated through prospective, visual, and ecological information gathering methods and particularly those which offer multiple modes of communication (e.g. body-map, visual, written, oral). Uptake of visual, ecological and prospective methods may enhance understandings of lived experiences of hallucinations.

*Funding:* University of Leicester.

**Research in context**

**Evidence before this study**

Systematic review prior to this 2018 study searched Web of Science Core Collection, PubMed and Medline for journal articles exploring hallucination experiences with search terms “hallucinat*” AND “phenomen*/”feel*”/”emot*”/”embodi*”, and “hearing voic*” AND “phenomen*/”feel*”/”emot*”/”embodi*”. Most articles retrospectively studied auditory verbal or visual hallucination phenomenology, with some considering bodily or emotional feelings. No identified articles had prospectively studied the immediate feeling of hallucinations: neither across modalities in an early intervention in psychosis sample nor using body-mapping.

**Added value of this study**

This study provides a more extensive dataset on the immediate feeling and embodied experience of hallucinations; with a novel contribution of body-maps and the prospective approach offering a higher level of ecological validity than is typically available. With existing research focused upon auditory hallucinations, this study provides novel insights into multimodal hallucinatory experiences of adults accessing early intervention in psychosis services.

**Implications of all the available evidence**

Immediate hallucination experiences appear co-constituted by feelings. Practice may be enhanced by offering alternative communication tools during information gathering (e.g. body-maps, visual diaries), providing interventions which address feelings shared across hallucination types (e.g. managing confusion, soothing anxiety, enhancing safety), and compassionately holding in mind the felt hallucination experiences people may be encountering.

Alt-text: Unlabelled box

## 1 Introduction

Hallucinations are a key area of psychiatric interest [1], and are characteristic of experiences described as psychosis and schizophrenia. Such experiences generate substantial costs to individuals and families through distress, isolation, and reduced relationships [2]. Contemporary psychiatric research has renewed interest in hallucination phenomenology, with aims to improve specificity of hallucinations considered characteristic of psychosis [3,4], such that concepts and taxonomies may be refined, causal pathways identified, and effective interventions developed.

Hallucination research has grown through substantial international collaborative efforts [5]. Mirroring historical descriptive psychopathology scholarship, contemporary research has focused upon auditory verbal hallucinations (AVH) – hearing voices. Phenomenological research has richly illustrated the multifaceted dimensions of AVH as varying in the extent to which they are: identifiable (in content, modality, quantity), characterful (in/animate features, natural/supernatural, linguistic and familiarity) imbued with relational feeling (communicated content, interactive, capable of influence, power, being known, and relationship) and feelings of reality (source and form, presence in consciousness, space and time) [3,4,[6], [7], [8], [9], [10]]. The sparse research into AVH's which considers this experience in terms of one's body (i.e. embodiment) has provided insights into hallucinations’ clinical features. As research continues to explore the lived experience of hallucinations, scholarship may be organised through utilising theoretical classifications of feelings including: emotional (i.e. sadness), extra-emotional/bodily (i.e. tiredness, pain), and of knowing (i.e. gut feelings) [11]. A further nuanced classification pertinent to hallucination studies may be feelings of reality; the extent to which experiences feel real and feelings of connection and disconnection to reality, oneself, one's body, and one's social-material circumstances in time and space.

The tone, timing, and content of hallucinations’ emotional feeling has been studied. In clinical populations, positive and negative emotionally toned AVH are reportedly commonplace [7]. AVHs emotional features are further indicated by reports that sadness, fear, anger, and loneliness are precipitative of AVH [6], with heightened stress described as causative of thought-like voices becoming audible for some [9]. Whilst hearing voices, emotions of depression and fear seem indicative of clinical populations, whereas broader feelings including positive and neutral responses are observed in non-clinical voice-hearers [4]. Notably, negative emotional valence of AVH content is estimated to distinguish current service-users with a psychotic spectrum diagnosis from healthy voice-hearers with 88% accuracy [10]. Identifying AVH's emotional features supports understanding lived experience and identifying clinical need.

Building upon the importance of emotional feelings, novel findings have arisen from studies of bodily feelings. In clinical populations, voices may vary in their volume, from whispered or soft (14%-31% of participants) through to conversational volume (35-73%) and loud, yelling, screaming or shouting (13-27%) [6,7]. In clinical AVH research, 45% of participants have reported butterflies or a churning stomach sensation before AVH or at onset [6], suggesting a sympathetic nervous system arousal response or anxiety. Early intervention in psychosis (EIP) service-users reported feelings of presence during AVH including imposition, force, being “held down”, and sensations of nausea, itching, and being given “physical pain” [3].^(p.93-94)^ In broader research, 66% of voice-hearers (clinical and non-clinical) report bodily feelings during AVH, including one's body feeling “on fire”, “more distant …dreamlike” and “tingling sensations throughout my extremities” [4]. ^(p.327)^ Participants reporting bodily feelings had distinct characteristics: better able to anticipate voices; voices were less positive or useful, more violent and abusive, more associated with shame; first voice experiences were more associated with traumatic circumstances [4].

Overall, research illustrates the relevance of emotional and bodily feelings to hallucinations, particularly auditory hallucinations (AH). Similar insights are emerging from studies of hallucinations of other kinds too. Akin to AVH, olfactory hallucinations (OH) are most often negatively emotionally valenced [12], and visual hallucinations (VH) have been reported with a range of precipitative feelings such as tiredness and loneliness [13]. Such research indicates the relevance of emotions and feelings across hallucinations modalities, perhaps multimodal hallucinations (MMH) may also be important to consider.

The relevance of MMH to clinical work has been empirically indicated for almost 50 years [6,14], with renewed interest in recent years with considerations of MMH's relevance, categorisation, clinical management and potential interrelationship with trauma [[15], [16], [17], [18]]. MMH involve two or more modalities and can either be simultaneous (multimodal at once) or serial (different modalities at different times) [19]. Although frequently neglected, contemporary phenomenological research has highlighted AVH's multimodal features [3,4]. Among people given a schizophrenia diagnosis, life-time prevalence studies indicate MMH may be the most common hallucination type [19]. Further studies of people given psychotic-spectrum diagnoses have also demonstrated the commonality of hallucinations beyond the auditory modality [20,21]. Such findings question the empirical justification of research and practice's focus towards unimodal AH, and suggest that unimodal and MMH involving OH, VH, tactile hallucinations (TH), and gustatory hallucinations (GH) require consideration.

Generating knowledge on dimensions such as modality, emotions and embodiment in a clinical sample can help further understanding of lived experiences of hallucinations and develop fitting methods of information gathering and intervention. To build on the literature, this study aimed to explore the question of how do hallucinations feel? Methodological scholarship has explored what methods fit questions such as these, with particular value arising from visual, arts-based, and body-mapping methods [22,23]. Outcomes of applying such methods in psychosis studies has been fruitful [3,24,25]. Existing methodological and clinical literature is built upon here by applying visual, diary, and body-mapping methods to explore the immediate feeling of hallucinations. To our knowledge, this is the first study of the immediate feeling of unimodal and multimodal hallucinations among EIP service-users. It is also the first study to body-map clinical hallucination experiences in everyday life.

## 2 Methods

### 2.1 Overview

The orientation of the study was based upon phenomenological hallucination research, process philosophy and qualitative methods [26]. The research question, methods and results reported here were a subsection of a larger study.

### 2.2 Participants

Participants were recruited from a community and outpatients NHS England EIP team serving an urban and rural region; Leicestershire and Rutland's Psychosis Intervention and Early Recovery Team. During routine clinical practice over seven months (May-December 2018), a purposive sampling strategy encouraged clinical practitioners to invite to the study service-users who were assessed through clinical interview as currently experiencing hallucinations (in any modality) and well enough to safely participate (recruitment criteria is shared in supplementary material Table S1). Participants were not known to the researcher (KM) prior to the study. The sampling strategy mirrors Upthegrove and colleague's strategy which argued studying EIP service-users enabled access to clinically relevant experiences close to service access and prior to longstanding clinical reframing which may alter participants own understandings of their experiences [3]. The sample size was guided by similar successful studies in the field [3,25], and to reduce sampling biases recruitment strategies encouraged recruitment of female and racialised participants.

Twelve participants chose to take part in this optional data generation stage (75% of the larger study's total sample [n=16]). Participants had an average age of 27.1 (SD=5.6), were all experiencing hallucinations daily and had been given psychotic spectrum diagnoses (supplementary material Table S2 displays further demographic data).

### 2.3 Procedures

For seven days prior to an interview, participants were asked to document hallucinations that arose within everyday life as soon as possible after they occurred using a structured paper diary. The diary was structured such that one diary page would document the experience of one hallucination. Together the data generated from each page is described as a multimodal unusual sensory experience (MUSE) map (unrelated to managing unusual sensory experiences trial) [27]. Each diary page had: tick box categories to indicate the involved modalities (e.g. visual, auditory, other etc); free text boxes to write and/or visually describe the sensory experience and feelings which arose; and a body-map to indicate what feelings were experienced and where. People could visually respond in the artistic medium of their choice; all chose to draw with pens or pencils. A total of 42 MUSE maps were generated (mean= 3 per participant, range 1-8).

The interview was face to face with the researcher (KM) in a clinic room and audio recorded via dictaphone. Participants were asked to describe their diary entries page by page and were invited to go into as much detail as possible. A semi-structured interview approach was taken such that participants were asked to provide further information for detail or clarification. Interviews lasted approximately thirty minutes and were transcribed verbatim.

Procedures were developed in collaboration with EIP multi-disciplinary staff, were positively reviewed by a service-user reference group and successfully piloted with two participants. The procedures built upon existing successful prospective diary AH and VH research [28], body-mapping approaches previously used to study the lived experience and felt emotion of psychosis [25,29,30], and photo-elicitation and diary methods studying AVH in EIP service-users [3]. Procedures were conducted by researcher KM, a female PhD candidate with academic research and clinical skills gained through experience and qualifications from undergraduate and postgraduate study, as well as working in NHS mental health services (including Psychological Wellbeing Practitioner Training, PGCert).

### 2.4 Participatory Analysis

A novel participatory embodied process analysis was used by KM under the supervision of the authors (JC, JC). The dataset for analysis was the MUSE maps, audio-recorded interviews and transcripts of all twelve participants. A participatory approach was employed, by using the interviews to ask participants own interpretations of their diary data and holding these as central throughout the interview and further analysis. Further analysis was completed via digital software. For written data and word cloud generation Microsoft Word and NVivo were used. For visual data, further programmes of Procreate and Adobe Illustrator were also used. Each participant's data was organised in relation to the subsections of the diary (i.e. modality, feeling, bodily feeling) and theoretical feeling categories [11], with researcher reflections added. Material was then collated to build interrelated understandings between participants (with particular interest in shared modality types). Overall, consistent with the research question the analysis explored the quality of experiences (qualitative methodology), with limited descriptive quantitative data offered to contextualise participants’ accounts.

To enhance quality, publishing guidelines for ensuring quality of qualitative research were followed [31,32]. With an independent researcher, coding classification of data was checked for a results subsection. The analysis was also reviewed by supervisors (JC, JC). The novel analytic approach stems from a history of qualitative methodological literature, which proposes the value of generating analytic strategies which fit the data rather than adopting off-the-shelf methods [33].

### 2.5 Ethics Statement

The research protocol and study materials were developed in accordance with the guidance from the NHS Health Research Authority (HRA) and Leicestershire Partnership NHS Trust Research and Development team. A successful research sponsorship application was made to the University of Leicester who reviewed the study materials and Integrated Research Application System (IRAS) form. After submission of the IRAS form, initial approval was provided by the NHS HRA and the study was put forward for NHS research ethics committee (REC) review. The London Camden and Kings Cross REC was attended and the study was reviewed by a panel of healthcare professionals and members of the public. Following positive review, ethical approval was granted (REC Identity Number: 18/LO/0418).

People interested in participating were provided with a study information sheet to read in full and opportunity to speak with the researcher (KM) and ask questions. All participants provided written informed consent to participate through reading and signing the informed consent sheet before taking part and were reminded of their right to withdraw their participation or data from the study.

### 2.6 Role of the Funding Source

University of Leicester. The funder had no role in design, data generation, analysis, interpretation, or writing. The final decision and responsibility for deciding to submit was held by the corresponding author (KM), who holds full access to study data.

## 3 Results

All twelve participants generated MUSE-maps via the diary and attended the follow-up interview. Hallucinations documented by participants included multimodal and unimodal variations of: non-verbal auditory hallucinations (NVAH), AVH, bodily hallucinations (BH), VH, OH, GH and disruptions in the feeling of time (Te). Supplementary material Table S3 illustrates all analytic sense categories were predominantly involved in MMH. AVH were the only unimodal experiences reported, although more participants reported AVH during MMH (a ratio of 1:5).

Analysis explored the extent of hallucination multimodality. The multimodality of diary data is shared in supplementary material Figure S1. Notably, 83% of participants (n=10) reported MMH which included both simultaneous and serial MMH. Participants reported experiences of MMH in three ways: serial only, simultaneous only, or serial and simultaneous. Reported by five participants, AH+BH were the most-commonly experienced modal form for each of these (with all but one AH being AVH). For participants reporting simultaneous MMH or both serial and simultaneous MMH, the modal picture was complex. Supplementary material Table S4 illustrates simultaneous MMH involving greater numbers of co-occurring modalities were experienced less often. BH and VH were present in many modal combinations (n=8 and n=7 respectively) and only slightly less common than AVH (n=9). Importantly, AH's greater frequency did not necessarily indicate greater subjective dominance of this sense within participants accounts. To summarise, participants reported hallucinations as arising in varied modalities, with a report-rate (highest-lowest) of: AH, BH, VH, TH, GH, OH and Te. Hallucinations typically involved multiple modalities either simultaneously or at separate time points.

**Figure 1:**
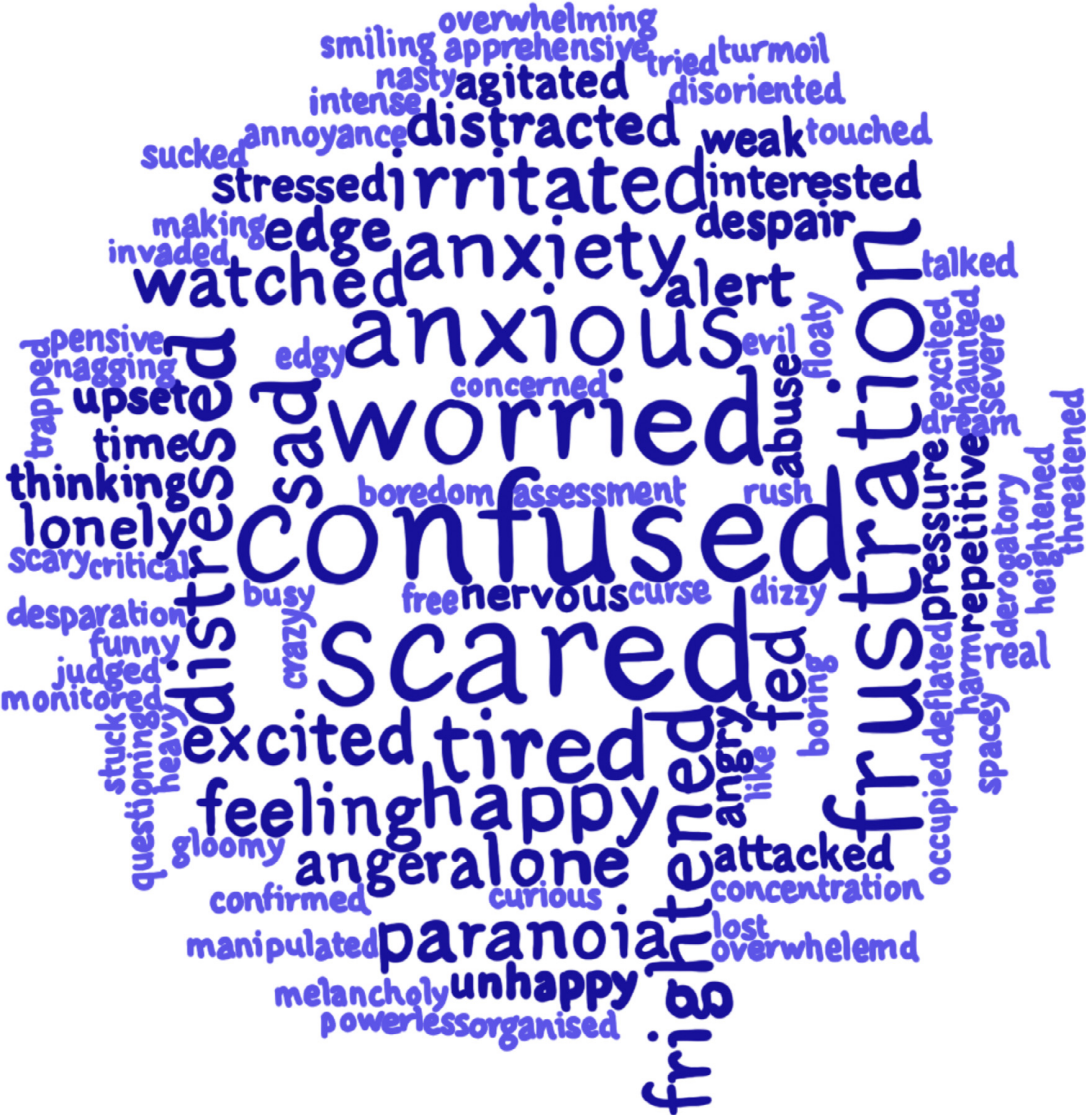
Quotes regarding the immediate feeling of hallucinations from participants during prospective data generation. The size of the text corresponds to the frequency, with larger text meaning the term was used more often.

Data from the “during this time I also felt” diary section, were screened for single word, or two word feeling terms; 106 terms were identified, with their corresponding frequency data informing the Figure 1 word-cloud for an overall picture of how hallucinations feel (the size of the words corresponds to their frequency). As each feeling is a participant quote, the word-cloud provides a picture of the immediate and difficult feelings characterising hallucinations. The feelings span theoretical feeling categories to include emotional feelings (“anxiety and frustration”), feelings of reality (“confusion”, “paranoia” and “manipulated”), feelings of knowing (“alert”, “overthinking”) and extra-emotional or bodily feelings (“tiredness”).

Emotional feelings were used to describe hallucinations by 92% (n=11) of participants. 41 terms were collectively used 111 times to describe the feeling of diary-documented hallucinations. Table 1 presents data on the immediate emotional feeling of hallucinations, including their frequency and co-occurring hallucination modalities. By order of frequency, the clusters of emotional feelings could be described as: fear and anxiety; despair and powerlessness; abused and threatened; frustration and anger; loneliness; stress and distress; worry; and positive feelings.

**Table 1:** Summary of the Frequency of Clusters of Emotional Feelings and the Modal types of Hallucinations they were Experienced within; Clusters Vertically ordered by Participant Numbers.

**Clusters of Feelings**	**H-N**	**P-N**	**F-N**	**Hallucination Types**
**Anxious, On-Edge, Scared, Frightened Nervous, Apprehensive**	35	9	6	AVH + BH + TH
				AVH + BH + VH
				OH + GH + TH
				AVH + BH
				VH
				AVH
**Powerless, Desperation, Despair, Turmoil, Trapped, Stuck, Worse, Wrong, Sad, Upset**	11	6	8	AVH + BH + VH + TH + GH
				AVH + BH + VH + TH
				AVH + BH + VH + Te
				AVH + VH + BH
				VH + BH
				AVH
**Abused, Attacked, Harm, Invaded, Touched, Manipulated, Uncomfortable, Vulnerable, Unpleasant, Threatened**	11	5	10	AVH + BH + VH + TH + GH
				AVH + BH + VH + TH
				AVH + BH + TH
				AVH + VH + BH
				AVH
**Frustrated, Irritated, Anger, Agitated, Annoyance, Wound-up**	22	4	6	AVH + BH + VH + TH + GH
				AVH + BH + VH +TH
				AVH + BH
				AVH
**Alone, Lonely**	7	4	2	AVH + BH + VH + TH + GH
				AVH + BH + VH + TH
				AVH + BH + VH
				AVH + BH + TH
				AVH
**Distressed, Stressed**	7	3	2	AVH + BH + VH + Te
				AVH + VH + BH
				AVH + BH
				VH + BH
				AVH
**Worried, Concerned, Pensive**	10	3	3	AVH + BH + VH + Te
				AVH + VH + BH
				OH + GH + TH
				AVH + BH
				VH + BH
**Happy, Excited, Funny, Smiling**	8	1	4	AVH + BH
				AVH
**Total**	111	11/	41	
		12		

Abbreviation Key: H-N: Number of times a feeling within a cluster was used to describe a hallucination.

P-N: Indicates the number of participants who diary documented a feeling.

F-N: Indicates the number of feeling terms.

AVH: Auditory-Verbal Hallucination BH: Bodily Hallucination VH: Visual Hallucination

TH: Tactile Hallucination,GH: Gustatory Hallucination, OH: Olfactory Hallucination, Te: Temporal Hallucination.

Feelings of knowing and reality were relevant too. Feelings of knowing were reported by 50% (n=6) participants, using 11 terms on 20 occasions as Table 2 summarises. These experiences pointed to potential hypoarousal of feeling “uninterested” and “bored” by hallucinations and conversely the hyperarousal of feeling “alert”, “pressure”, “busy”, and “overthinking”. Feelings of reality were reported by 92% (n=11), using 31 feeling terms on 49 occasions to describe hallucinations. As Table 3 summarises, some feelings of reality regarded: watchful entity qualities of hallucinations and their malevolent atmosphere; disorientation and disconnection; the confusing and occupying pull of hallucinations; the paranoia and overwhelming feeling of experiencing and making sense of it all. Feelings of reality were experienced in hallucinations of each modality documented within the study. As hallucinations are defined by difference to consensual reality, it makes sense that feelings of reality would be common.

**Table 2:** Summary of the Frequency of Clusters of Feelings of Knowing and the Modal Types of Hallucinations they were Experienced within.

**Clusters of Feelings**	**H-N**	**P-N**	**N-F**	**Hallucination Types**
**Uninterested, Bored, Repetitive, Nagging**	7	2	4	AVH + VH + BH +TH
				AVH
**Pressure, Overthinking, Busy, Rushing, Organised**	7	3	4	AVH+BH
				AVH
				VH
**Alert, Concentration**	6	3	3	AVH + GH + BH
				AVH + BH
				AVH
**Total**	20	06/12	11	

Abbreviation Key: H-N: Number of times a feeling within a cluster was used to describe a hallucination. P-N: Number of participants who diary documented a feeling. F-N: Number of feeling terms per cluster.

AVH: Auditory-Verbal Hallucination, BH: Bodily Hallucination, VH: Visual Hallucination, TH: Tactile Hallucination, GH: Gustatory Hallucination.

**Table 3:** Summary of the Frequency of Clusters of Feelings of Reality and the Modal Types of Hallucinations they were Experienced within.

**Clusters of Feelings**	**H-N**	**P-N**	**F-N**	**Hallucination Types**
**Nasty, Evil, Haunted, Curse, Derogatory**	5	5	5	AVH + VH +GH + BH + TH AVH + BH VH + BH AVH
**Disoriented, Dizzy, Dreamlike, Floaty, Spacey, Zoned-out,**	6	4	6	AVH + VH + BH +TH AVH + BH AVH BH
**Confused, Uncertainty, Unsure**	12	4	3	AVH + VH + BH +TH AVH + GH + BH AVH + BH + TH AVH + BH
**Watched, Under-Assessment, Judged, Monitored,** **Talked-About**	8	3	5	AVH + VH + BH AVH + BH AVH
**Distracted, Occupied**	4	3	2	OH + GH + TH AVH + BH AVH
**Real, Lost-Time, Confirmed**	3	3	3	AVH + VH + BH+ Te AVH + BH
**Curious, Questioning, Critical**	3	3	3	AVH + GH + BH AVH + BH NVAH
**Overwhelmed, Intense**	3	3	2	AVH + VH + BH +TH AVH + BH + VH + Te AVH + BH AVH
**Paranoia**	4	2	1	AVH + BH + TH AVH + BH AVH
**Free**	1	1	1	AVH + BH
**Total**	49	11/ 12	31	


Abbreviation Key: H-N: Number of times a feeling within a cluster was used to describe a hallucination. P-N: Number of participants who diary documented a feeling. F-N: Number of feeling terms per cluster. AVH: Auditory-Verbal Hallucination BH: Bodily Hallucination VH: Visual Hallucination TH: Tactile Hallucination GH: Gustatory Hallucination OH: Olfactory Hallucination Te: Temporal Hallucination

**Figure 2:**
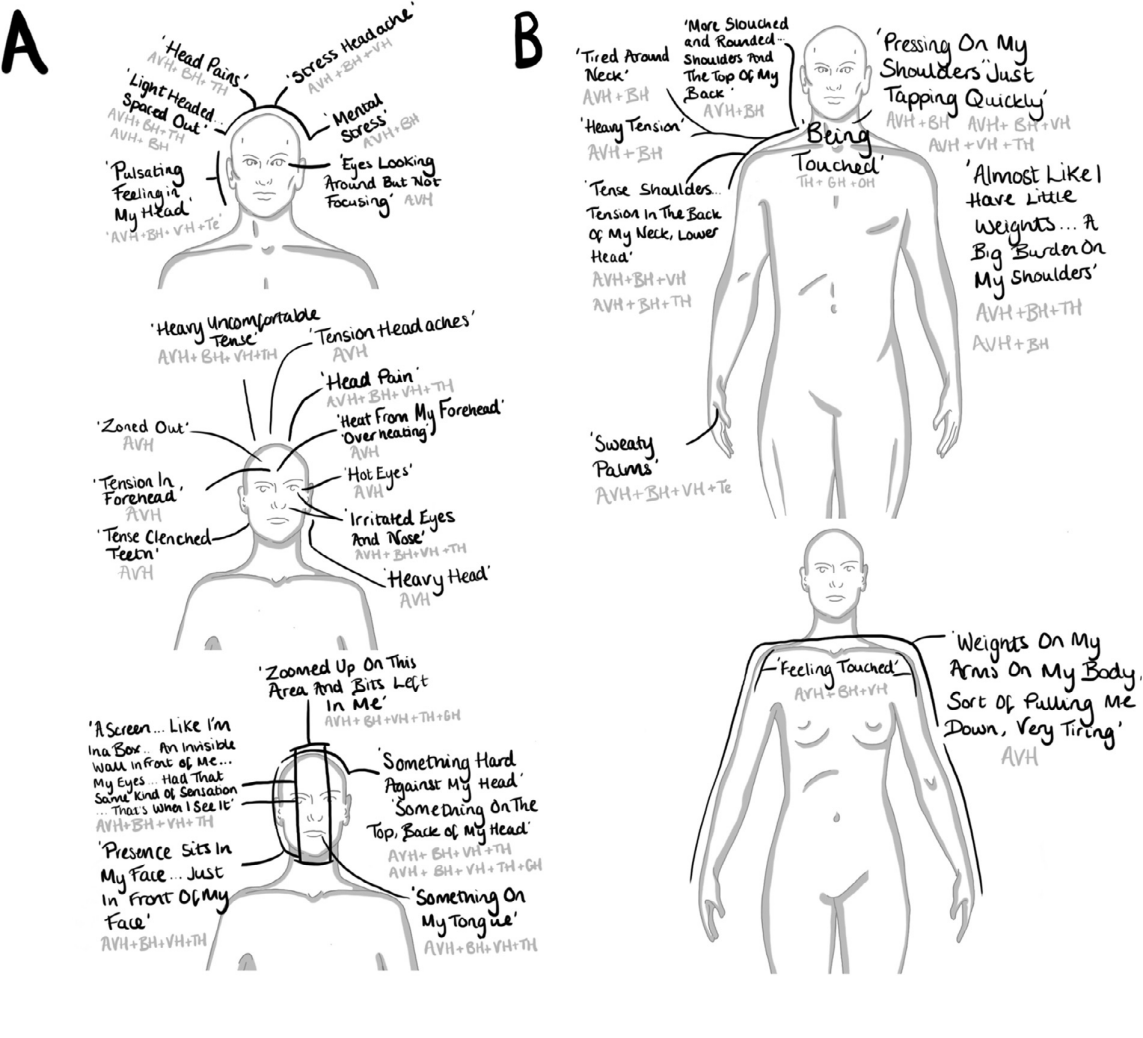
Illustrations of collated body-map data localised to the head (A), and to the neck, shoulders and arms (B). Black writing holds participants' quotes, grey writing shares the co-occurring hallucination modality, + symbols indicate multimodality. Abbreviations: auditory verbal hallucinations (AVH), bodily hallucination (BH), visual hallucination (VH), tactile hallucination (TH), gustatory hallucination (GH), and temporal (Te).

**Figure 3:**
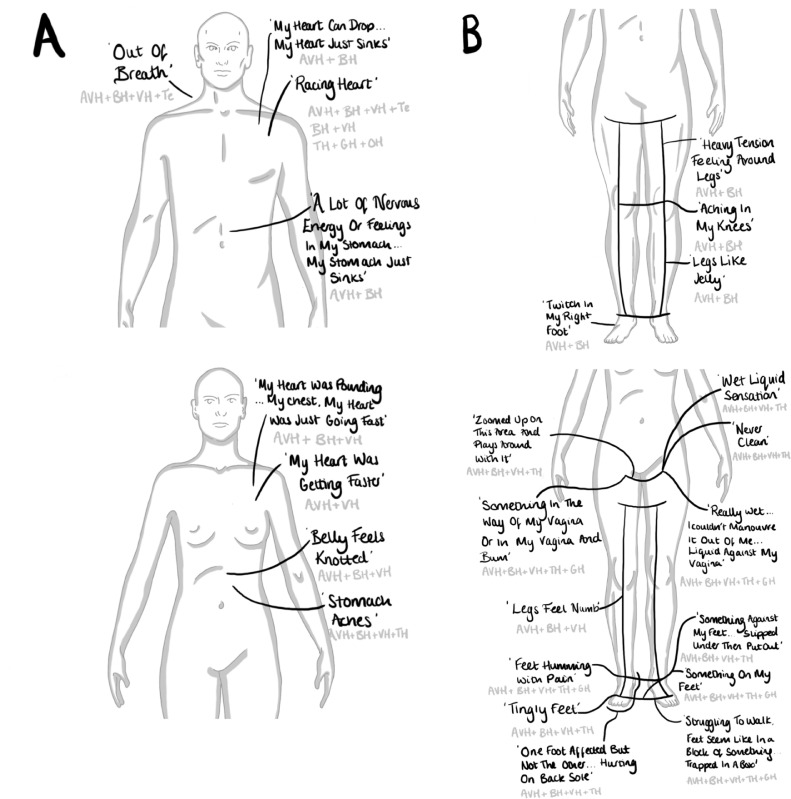
Illustrations of collated body-map data localised to the chest and abdomen (A), and to the pelvis, legs and feet (B). Black writing holds participants' quotes, grey writing shares the co-occurring hallucination modality, + symbols indicate multimodality. Abbreviations: auditory verbal hallucinations (AVH), bodily hallucination (BH), visual hallucination (VH), tactile hallucination (TH), gustatory hallucination (GH), olfactory hallucination, and temporal (Te).

All participants reported bodily feelings and sensations as co-occurring with hallucinations and most participants (75%, n=9) reported experiencing BH. This analysis section presents digital body-maps. The researcher collated and digitally regenerated participants’ body-map data and hallucination types to illustrate examples of: what feelings were experienced, where in the body feelings arose, and during what kind of hallucinations. To support analytic specificity, body-map data was illustrated separately for participants self-identifying as male or female. During analysis, reported bodily feelings were analytically categorised as localised to an identifiable body part or generalised across the body. The body-maps illustrate feelings localised to the: head (Figure 2a), neck, shoulders, back and arms (Figure 2b), chest and abdomen (Figure 3a), and pelvis, legs and feet (Figure 3b).

The body-map data suggested a variety of co-occurring bodily feelings across hallucination types, and that these feelings can be identifiable, localised, specific, and communicable. The head and shoulders seemed to be the areas where feelings were localised most-often for participants and arose across hallucination types. The chest, abdomen, legs, and feet were also areas where feelings seemed concentrated. No male participants documented feelings within their genitals or pelvis; in contrast, for one female participant, many feelings were localised to these areas. The hands and back were not frequently documented sources of localised bodily feelings.

**Figure 4:**
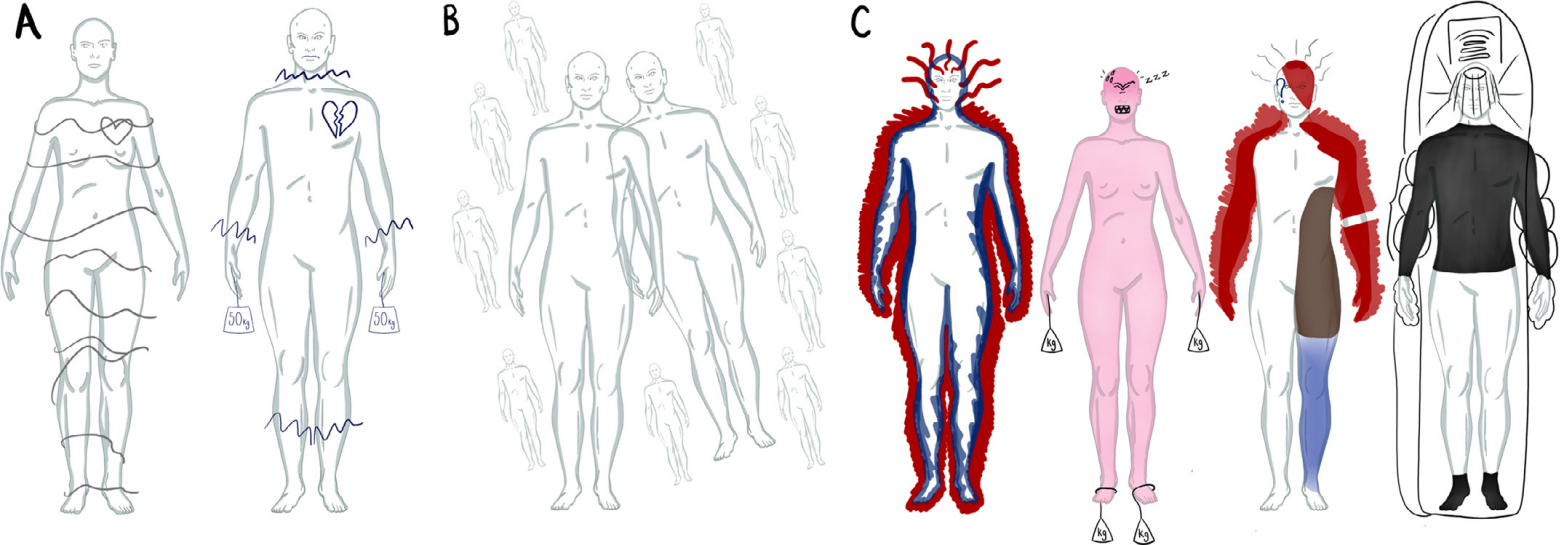
Digitally regenerated body-maps illustrating the immediate feeling of hallucinations involving one sense. Body-maps provide visual illustrations of both the embodiment of the feeling of hallucinations involving one sense at the time, and the extension of feeling beyond the body into peripersonal space. The form and colour of the markings on the body-maps were illustrated by participants and digitally regenerated by the researcher (KM). 4a: From left to right: visual hallucination (one body-map from one participant) and bodily hallucination (collation of two body-maps from one participant). 4b: Providing an example of one body-map from one participant which illustrates auditory verbal hallucination (one body-map from one participant). 4c: From left to right: Auditory verbal hallucination (collation of two participant's body-maps), auditory verbal hallucination (collation of seven body-maps, six from one participant, one from another), auditory verbal hallucination (collation of two body-maps from one participant) and auditory hallucination (collation of seven body-maps, six from one participant, one from another).

**Figure 5:**
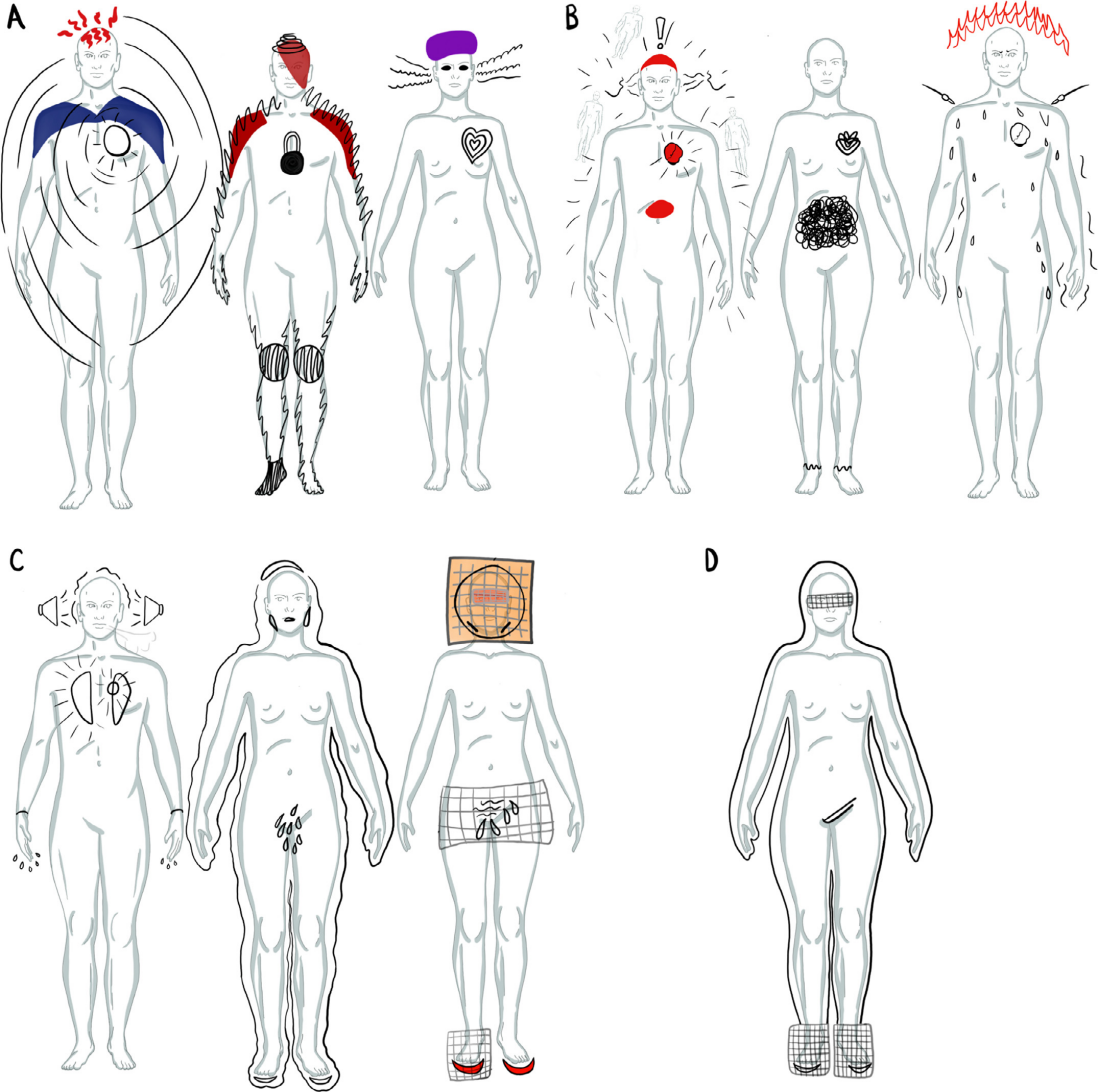
Digitally regenerated body-maps illustrating the immediate feeling of simultaneous multimodal hallucinations. Body-maps provide visual illustrations of both the embodiment of the feeling multimodal hallucinations, and the extension of feeling beyond the body into peripersonal space. The form and colour of the markings on the body-maps were illustrated by participants and digitally regenerated by the researcher (KM). 5a: From left to right: auditory verbal and bodily hallucination (collation of two participant's body-maps), auditory verbal and bodily hallucination (collation of two participant's body-maps), auditory verbal and visual hallucination (one participant's body-map). 5b: From left to right: auditory verbal, bodily and visual hallucination (collation of two participants' body-maps), auditory verbal, visual and tactile hallucination (one participant's body-map), tactile, gustatory and olfactory hallucination (one participant's body-map). 5c: From left to right: auditory verbal, visual, tactile and temporal hallucination (one participant's body-map), auditory verbal, bodily, visual and tactile hallucination (one participant's body-map), auditory verbal, bodily, visual and tactile hallucination (collation of one participant's three body-maps). 5d: From left to right: auditory verbal, bodily, visual, tactile and gustatory hallucination (one participant's body-map).

During hallucinations of specific modal kinds, through body-mapping participants shared the body areas in which feelings were experienced. To support data presentation, Figure 4, Figure 5 share examples of digital regenerations of body-maps which were collated: each figure caption provides collation details. Body-maps are presented in order of one to five simultaneous modalities. The body-maps illustrate feelings may be experienced across many different body areas during a single instance or type of hallucination. Although there was great variation in the localisation of feelings across the sample, for each participant feelings were recurrently concentrated in particular body areas. Areas of concentration often held repeated sources of feelings like pain, heat, or tension during hallucinations of different modal kinds. Alongside these feelings localised within specific body areas, these body-maps illustrate how generalised feelings across the body were reported too.

Generalised feelings experienced across the body are summarised in Table 4. Some of the feelings reported here as generalised were described as localised by other participants; for example feeling tense throughout the body versus tension in the neck. There were broader feelings such as “adrenaline”, “exhaustion”, or “watched” which although identifiable within the body, were generalised across the body in a way that seemed distinct from those analytically described as localised. Notably many body-maps illustrated that the subjective felt experience of hallucinations is both felt within the body and peripersonal space. This aspect emphasises the ways in which hallucinations may enmesh ones internal and external worlds.

**Table 4:** Summary of the Frequency of Clusters of Bodily (Extra-Emotional) Feelings and the Modal Types of Hallucinations they were Experienced within.

**Clusters of Feelings**	**H-N**	**P-N**	**F-N**	**Hallucination Types**
**On-edge, Tense, Pressure, Fight or Flight, Adrenaline, Agitated**	12	8	6	AVH + BH + VH +TH AVH + BH + VH AVH + BH + GH AVH + BH AVH VH
**Tired, Heavy, Weak, Exhaustion, Knackered, Weighted, Struggling, Solid**	16	7	8	AVH + VH + BH +TH AVH + BH + VH AVH + BH AVH + VH AVH BH
**Hot, Cold, Sweaty, Chills**	6	3	4	AVH + BH + GH TH + GH + OH AVH + BH VH + BH BH
**Wobbly, Dizzy, Shaky, Jumpy, Light, Tingly, Trembling**	5	3	7	AVH + BH + TH + GH + OH AVH + VH VH + BH AVH VH
**Daren't move, I Couldn't Move because my Body was too in Shock, Stuck**	3	2	3	AVH + BH + VH VH + BH VH
**Energised, Conscious Energy**	2	2	3	AVH + BH AVH
**Unresponsive, Not Real, Disconnected, Like Air, Not in Myself just Watching Myself**	2	1	5	BH AVH
**Something Against my Body All Over…Placed On the Top. It Feels like a Heat**	2	1	2	AVH + BH +VH + TH + GH AVH + BH + VH +TH
**Subtle pain, Severe Discomfort**	1	1	2	AVH + VH + BH +TH
**Feeling of Someone Watching**	1	1	2	AVH + VH
**Wet**	1	1	1	AVH + BH + VH + TH
**Relaxed, Kind of High**	2	2	2	AVH + BH + VH AVH + BH
**Like There was Something Inside of me Pushing Outwards**	1	1	1	AVH + BH
**Total**	54	11/12	46	

Abbreviation Key: H-N: Number of times a feeling within a cluster was used to describe a hallucination. P-N: Number of participants who diary documented a feeling. F-N: Number of feeling terms. AVH: Auditory-Verbal Hallucination BH: Bodily Hallucination VH: Visual Hallucination TH: Tactile Hallucination GH: Gustatory Hallucination OH: Olfactory Hallucination.

An important result to note, is the simultaneous co-occurrence of feelings of varied types (modal, emotional, bodily [localised/generalised], knowing, reality) during hallucinations. This co-occurrence of feelings, although highly variable, typically characterised reports of the immediate experience of hallucinations. Supplementary Figure S2 illustrates the immediate experience of an MMH of BH+TH+AVH+VH (in order of sensory dominance); visually mapping what feelings arise, co-occur and where they're felt in relation to the body. This highlights the complex subjective experience people may be navigating which may not be readily identifiable by or shared with those around them.

## 4 Discussion

This study provided the most extensive descriptive data to date on the immediate feeling of hallucinations across modalities in EIP service-users (in terms of sensory, emotional and embodied feelings) and the experience of bodily sensations as part of hallucinatory experiences using a novel MUSE map method. The outcomes chimed with existing research through substantiating the relevance of emotional and bodily feelings to hallucination experiences in clinical populations [6,7,30,34]. It built upon such evidence through identifying the location and content of various feelings during hallucinations; including feelings localised or generalised within the body and extensions into peripersonal space. It also builds upon existing phenomenological research, by providing novel prospective data of hallucinations as they are lived in daily life rather than retrospective accounts at a single time-point.

Usually hallucinations are studied and reported upon with a unimodal focus, with MMH considered as less common [4,9,13], within the current study however MMH were prominent among 83% (n=10) of participants. This prominence of MMH substantiates contemporary prevalence research which has suggested MMH are common [19]. The current study's findings also chime with mid-20th century studies, that emphasised MMH were commonplace and characteristic experiences of people described as paranoid or given schizophrenia diagnoses, and that these experiences were less readily communicated and under-recognised [14]. Based on the data and the growing corpus of outcomes on the embodied features of hallucinations, it is reasonable to propose hallucinations may often be characterised by multimodality.

Usually MMH research has emphasised AH+VH combinations [13,19], but in the current study AH+BH were most common. This outcome is consistent with Woods and colleagues’ research [4],^(p.326-327)^ where 18-28% reported MMH but a much higher 66% reported co-occurring bodily feelings whilst hearing voices. With feelings often being fleeting and passing by unnoticed [11], perhaps the prospective MUSE map method and broader conceptualisation of hallucinations supported in generating this study's novel AH+BH outcome. The utility of this study's methods in generating data on the sensory, emotional and embodied aspects of hallucinations, may have clinical and research implications for information gathering. Perhaps broader concepts and information gathering methods such as those used here (visual, ecological and prospective) may overcome some of the difficulties service-users and clinicians encounter when trying to talk about hallucinations [35,36].

Previous retrospective studies signalled the relevance of broader feelings and embodiment in hallucinations [4] and the localisation of emotional feelings within psychosis [30]. Existing research is built upon in this study which offers accounts of hallucinations in numerous modalities alongside prospective data on what feelings and emotions are felt during hallucinations, and where these are felt. Novel outcomes included the visual description of hallucinations, illustrations of co-occurring feelings and lastly, that documented bodily feelings extended beyond the body into peripersonal space; an outcome which may have implications for understanding experiences of presence and paranoia.

Where retrospective research reported two thirds of voice-hearers experiences were accompanied by bodily sensations [4], in this prospective clinical study, all documented hallucinations co-occurred with bodily feelings. Some feelings reported in existing research (like nausea, itching, feeling on fire and shock-like sensations in one's chest) were not reported [3,4]. Other feelings reported did however chime with existing research. Similar to AVH, documented in EIP service-user research, participants in the current study also reported experiences of: “pains”, “pressing on my shoulders”, “tension in the back of my neck, lower head” and “being touched” on shoulders [3]. Further AVH research similarities included reported sensations of “heat from my forehead”, “tingly feet” and one's “belly” feeling “knotted” [4,6]. In previous studies such bodily feelings were discussed in terms of experiences described by researchers as AVH or voice-hearing, however in the current study (where participants themselves documented modality prospectively) these quoted sensations arose within simultaneous MMH. The novel outcomes suggest lived experiences of hallucinations may diverge from top-down concepts researchers and clinicians bring. Attending to such nuances in lived experience may help inform the ongoing development of hallucination-specific interventions [27,37].

Clinical implications of the current study include: adapting communication; adjusting practice to consider sensory, embodied and emotional needs; trauma-informed practice and enhancing safety. Adapting communication in clinical practice to generate rich person-centred accounts may include offering options of visual (e.g. drawing), prospective (e.g. diary), and body-focused (e.g. body-mapping) methods when gathering information in clinical practice (e.g. at assessment, review or during interventions). Consideration of sensory, embodied and emotional needs can be aided by adapting communication and further factors. Firstly, it may be pertinent for practitioners to keep in mind that complex feeling experiences may be arising during interactions; to acknowledge the potential for this, offer empathetic exploration of these where relevant and provide collaborative support to help people feel as safe as possible. Secondly, practitioners should hold an open mind regarding the sensory and embodied feeling of hallucinations and be aware of biases towards favouring or focusing upon unimodal and auditory experiences. Thirdly, modifications to clinical environments may aid sensory needs and practitioners can ask what adjustments to their approach (e.g. their pacing) or adjustments to setting (e.g. quiet rooms) may help. Adjustments in practice may include uptake of interventions which attend to embodiment and soothing (e.g. breathing meditations, movement-based interventions, body-mapping, art, music) and incorporating existing coping strategies already in place.

The importance of attending to embodiment, feelings, safety, and choice in clinical practice is consistent with trauma-informed care approaches. Broader psychiatric literature is increasingly learning how “the body keeps the score” of the impact our lived circumstances, adversities and traumas [38]. Alongside accommodating for the potential contribution of past trauma, further clinical implications include managing the safety of current circumstances. Clinical implications may include making clinical services as safe as possible and promoting efforts to increase safety beyond the clinic room too; for example, through supporting applications for safe housing, or utilising professional power to advocate for trauma prevention.

Associated with the current study is possible risk of clinical misapplication, and three methodological limitations. A focus towards multimodality and embodiment of hallucinations may generate diagnostic overshadowing risks. Bodily and sensory feelings indicative of harmful physiological processes could be wrongly understood as part of hallucinatory experience. This study illustrates hallucinations may often be perceived as bodily experiences and provides examples of what feelings may co-occur for people during hallucinations, allowing such experiences to be more fully understood and addressed clinically. In clinical practice, it is critical that bodily feelings and sensations are appropriately medically assessed before psychiatric and psychological conceptualisations are applied to them. Nuanced interpretations of feelings may involve considering dimensions of the feelings (e.g. intensity, duration, frequency, modifiers, onset) and their relationship to medication side-effects, indicators of health and illness, and potential interrelationship with hallucinations.

Regarding this study's exploratory nature and methods, akin to existing phenomenological research there may be underrepresentation of some experiences (e.g. pain) because participatory and visual methods place agency on participants to guide what information is relevant and communicated. Woods and colleagues emphasised that to generate a fitting psychiatric knowledge base, phenomenological studies and methods of subjective experience “are a necessary starting point” upon which standardised methodologies can build. ^4(p.330)^

A second limitation regards the diary micro-body-map. Although it could be interpreted as a body front or back, the body-maps were mostly used as front-facing. Future research may benefit from holding two body-maps (titled ‘front’ and ‘back’) or 3D modelling.

A third limitation regards potential generalisability due to sample size and locality, as a small sample was recruited from a single site. Nevertheless, this site spans a diverse geographical, cultural and socio-economic region and allowed empirical data generation by a single researcher, which facilitated closeness to the data and a consistent participatory ethos. Although the sample's size and homogenous features are fitting with phenomenological and visual qualitative studies [4], there may be limitations in results generalisability.

This study generated clinically-relevant insights which may inform changes in practice, with implications for clinicians and researchers. Overall, the novel methodology, focus upon feeling, and prospective study of hallucinations across modalities in EIP service-users provided insights which built upon contemporary scholarship and provided novel examples. These outcomes indicate co-occurring bodily feelings contribute to hallucinations’ immediate experience alongside the potential prominence of MMH and difficult feelings (emotional, of reality, of knowing). This study provides a further example of the relevance of visual and arts-based approaches to hallucinations, which is consistent with their growing uptake in research methods and practice [3,25,37,39].

Further research may benefit from building upon the current design to study immediate felt experience of hallucinations, whilst gathering perspectives of why these feelings occur and what would help in preventing, communicating and managing distressing experiences. Further research may also be enhanced through the development of a quality standards checklist for visual and participatory clinical research, and the triangulation of methodologies (e.g. clinical notes review, or ecological methods such as experience sampling). For clinical practice, key implications include utilising prospective and visual information gathering methods; identification and normalisation of multimodal, felt and somatic features of hallucinations; utilising evidenced-based artistic, body, and music-based interventions; [40] trauma-informed care; and nuancing interpretations of somatic experiences in EIP populations. This study's outcomes illustrate the value of investigating lived experience such that we can approach people's needs with fitting concepts, methods of information gathering, and interventions.

## Contributors

Dr Katie Melvin was the Lead Researcher and Author: Systemic Literature Review Lead, Study Design Lead, Ethics Approval Lead, Sole Data Collector, Data Analysis Lead, Data Interpretation Lead, Lead Author, Sole Illustrator.

Dr Jon Crossley and Prof John Cromby (Equal Contribution) provided supervision for Dr Katie Melvin and Supported in: Developing Research Idea, Systematic Literature Review Interpretation, Developing Study Design, Preparing for and Seeking Ethical Approval, Data Analysis and Interpretation, Editing and Refining Writing.

Dr Jon Crossley: Principal Investigator at Clinical Site and Supported the Data Collection by Linking the Study with the Service and Practitioners, Ensuring study Feasibility and Safety.

## Funding

University of Leicester.

## Data Sharing Statement

Due to the nature of this research, no additional data are available upon reasonable request; participants of this study did not agree for their datasets to be shared.

## Declaration of Competing Interest

All the authors report no conflicts.

## Appendix Supplementary materials

Image, application 1
