# Supplementary material for: The feeling, embodiment and emotion of hallucinations in first episode psychosis: A prospective phenomenological visual-ecological study using novel multimodal unusual sensory experience (MUSE) maps
Source: eClinicalMedicine. 2021 Oct 16;41:101153. doi: 10.1016/j.eclinm.2021.101153 (PMC8633969; doi:10.1016/j.eclinm.2021.101153)
Supplement: Supplementary file 1 [file mmc1.docx]

# Supplementary Material

# Figures

Figure S1: Proportion of Participants Reporting Unimodal or Multimodal Hallucinations (MMH).


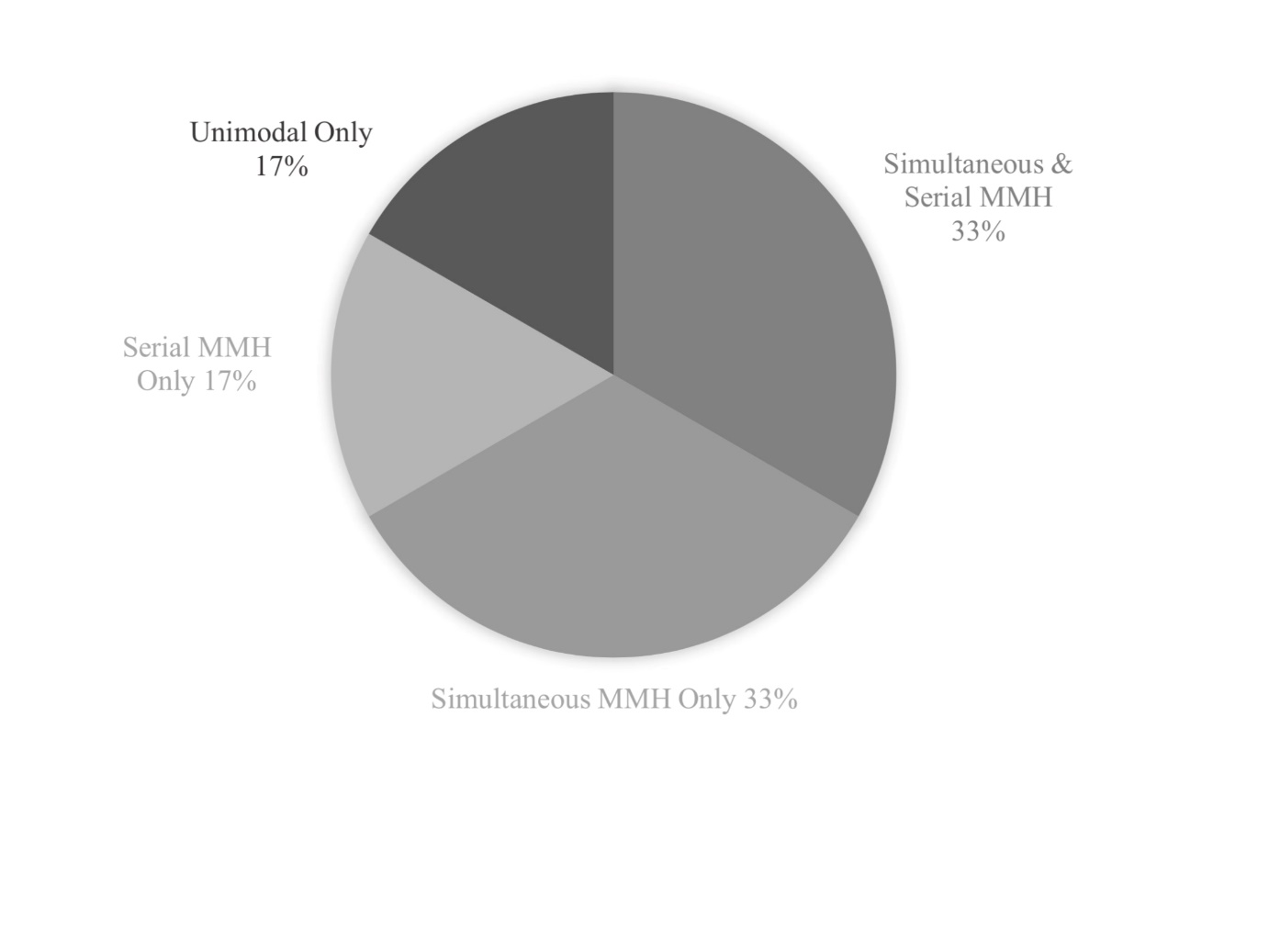


Figure S1: Summary of modal types of hallucinations as reported by participants during prospective data generation.

Figure S2: A multimodal unusual sensory experience (MUSE) map of the immediate feeling of simultaneous multimodal hallucination.


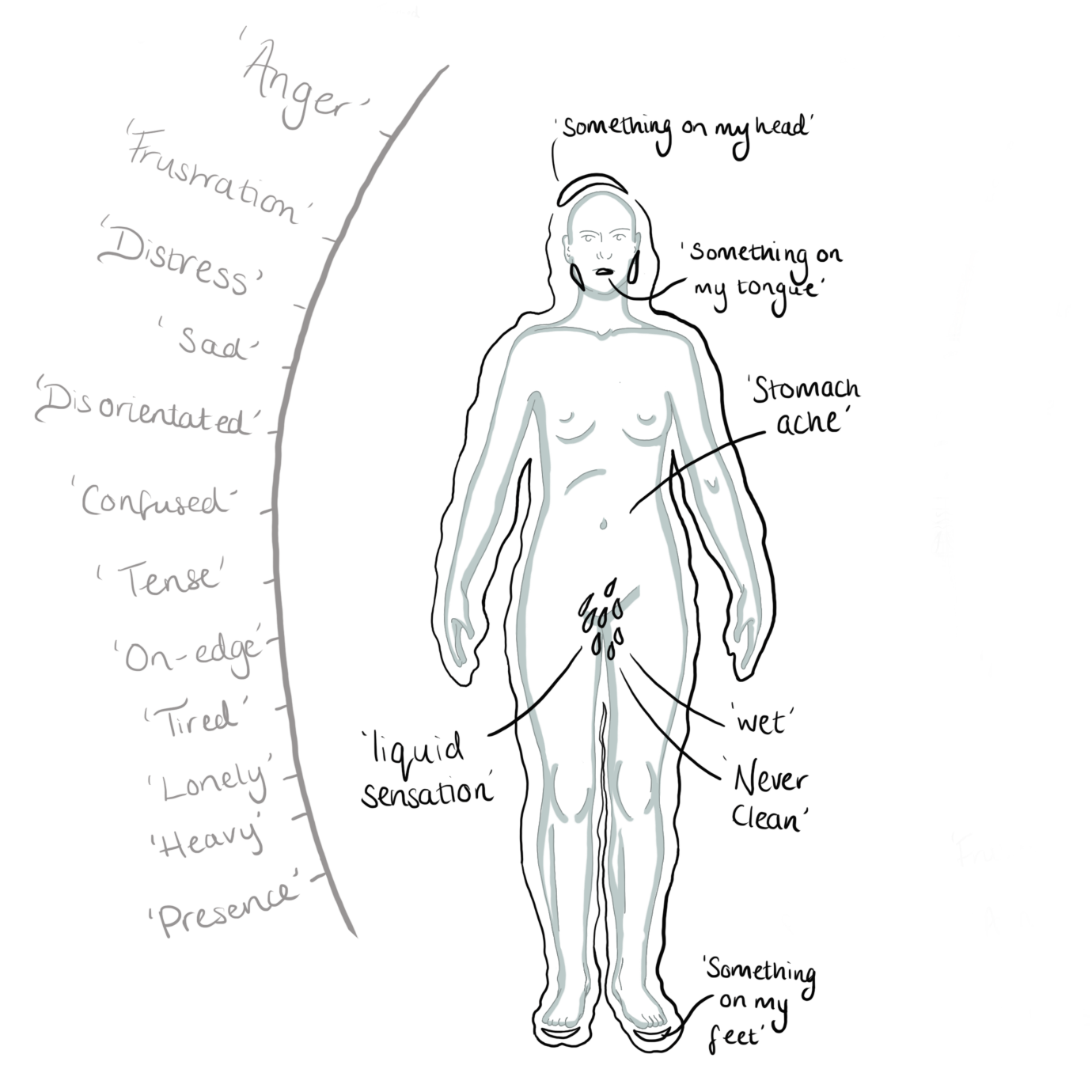


Figure S2: This is a multimodal unusual sensory experience (MUSE) map focused on the feeling data, which collates participant’s visual, written and auditory data regarding a hallucination experience (as documented in the visual diary and further described during semi-structured interview). The black writing regards the participant’s quotes regarding her body-map, the grey writing around the side holds her further quotes of co-occurring feelings during this hallucination experience. The MUSE map illustrates that the participants immediate hallucination experience involved feelings which can be described as embodied, peripersonal, emotional, of reality, of knowing and sensory (auditory verbal, bodily, visual, tactile and gustatory hallucination).

# Tables

Table S1: Recruitment inclusion and exclusion criteria.

| **Table S1: Recruitment inclusion and exclusion criteria.** | |
| --- | --- |
| **Criteria** | **Requirements for Recruitment.** |
| **Inclusion** | Currently accessing early intervention in psychosis service. |
| **Inclusion** | Aged 18-64 (early intervention in psychosis [EIP] service age range). |
| **Inclusion** | Perceived by their care coordinators as having the capacity to consent to and participate in the research. |
| **Inclusion** | Participants must be perceived by their care coordinators as able to safely engage with the data collection procedures. This includes but is not limited to, being able to safely manage any potential distress associated with discussing their hallucinations and personal histories and being safe to be in an EIP clinic room with the researcher (i.e. not being a risk to the researcher). |
| **Inclusion** | Able to complete the interviews and diary in English comfortably. |
| **Inclusion** | Participants can be of any gender identity. |
| **Exclusion** | Experiencing hallucinations at a less regular frequency than every other day. |
| **Exclusion** | Hallucinations known to be due to: another health condition, current medication use or current substance use. |

Table S2: Demographics of participants currently experiencing hallucinations daily who completed the visual diary.

| **Table 2: Demographics of participants currently experiencing hallucinations daily who completed the visual diary.** | | |
| --- | --- | --- |
| **Age** | | |
| **18-25** | **26-40** | |
| 5 (42%) | 7 (58%) | |
| **Gender** | | |
| **Male** | | **Female** |
| 8 (67%) | | 4 (33%) |
| **Ethnicity** | | |
| **White** | **Indian** | |
| 7 (58%) | 3 (25%) | |
| Black Afro-Caribbean | Mixed Black African and White European | |
| 1 (8%) | 1 (8%) | |
| **Religiosity** | | |
| **Religious** | **Non-Religious** | |
| 6 (50%) | 6 (50%) | |
| Religious= Hindu (n=2), Sikh (n=1), Muslim (n=1), Christian (n=1), Multifaith (n=1) | | |
| **Diagnosis** | | |
| **Schizophrenia** | **Other Psychotic Spectrum Diagnosis** | |
| 7 (58%) | 5 (42%) | |
| **Currently Taking Anti-Psychotic Medication** | | |
| **Yes** | **No** | |
| 7 (58%) | 5 (42%) | |
| Anti-Psychotic Medications= Quetiapine (n=3), Lurasidone (n=2), Aripiprazole (n=1), Haloperidol (n=1) | | |

Table S3: Summarising the Modality Involvement in Unimodal and Multimodal Hallucinations (MMH).

| **Table S3: Summarising the Modality Involvement in Unimodal and Multimodal Hallucinations (MMH)*.*** | | | | | | |
| --- | --- | --- | --- | --- | --- | --- |
| **Hallucination Modality** | **Unimodal** | **Serial MMH** | | **Simultaneous MMH** | | **Total** |
|  | **P-N** | **M-N** | **P-N** | **M-N** | **P-N** | **P-N** |
| **Auditory (Non-Verbal)** | .. | 1 | 1 | .. | .. | 1 |
| **Auditory (Verbal)** | 2 | 1 | 1 | 10 | 9 | 12 |
| **Bodily** |  | 2 | 2 | 8 | 7 | 9 |
| **Visual** |  |  |  | 7 | 4 | 4 |
| **Tactile** |  |  |  | 6 | 4 | 4 |
| **Gustatory** |  |  |  | 3 | 2 | 2 |
| **Olfactory** |  |  |  | 1 | 1 | 1 |
| **Temporal** |  |  |  | 1 | 1 | 1 |
| P-N: Number of participants who experienced hallucinations in that modality. M-N: Number of multimodal hallucination types the sensory modality was involved in. | | | | | | |

Table S4: Modality Combinations of Simultaneous Multimodal Hallucinations: Horizontally and Vertically Ordered by Frequency of Modality Involvement.

| **Table S4: Modality Combinations of Simultaneous Multimodal Hallucinations: Horizontally and Vertically Ordered by Frequency of Modality Involvement**. | | | | | | | | | | |
| --- | --- | --- | --- | --- | --- | --- | --- | --- | --- | --- |
| **Participant N** | **Modalities** | | | | | | | | | |
| 5 | AVH | 🡨🡪 | BH |  |  |  |  |  |  |  |
| 1 | AVH | 🡨🡪 | BH | 🡨🡪 | VH |  |  |  |  |  |
| 1 | AVH | 🡨🡪 | BH | 🡨🡪 | VH | 🡨🡪 | Te |  |  |  |
| 1 | AVH | 🡨🡪 | BH | 🡨🡪 | VH | 🡨🡪 | TH |  |  |  |
| 1 | AVH | 🡨🡪 | BH | 🡨🡪 | VH | 🡨🡪 | TH | 🡨🡪 | GH |  |
| 1 | AVH | 🡨🡪 | BH | 🡨🡪 | TH |  |  |  |  |  |
| 1 | AVH | 🡨🡪 | BH | 🡨🡪 | GH |  |  |  |  |  |
| 1 | AVH | 🡨🡪 | VH |  |  |  |  |  |  |  |
| 1 | AVH | 🡨🡪 | VH | 🡨🡪 | TH |  |  |  |  |  |
| 1 | BH | 🡨🡪 | VH |  |  |  |  |  |  |  |
| 1 | TH | 🡨🡪 | GH | 🡨🡪 | OH |  |  |  |  |  |
| Colour coded table with a colour per modality to aid reading of the table. | | | | | | | | | |  |
